# Predicting Physical Appearance from DNA Data—Towards Genomic Solutions

**DOI:** 10.3390/genes13010121

**Published:** 2022-01-10

**Authors:** Ewelina Pośpiech, Paweł Teisseyre, Jan Mielniczuk, Wojciech Branicki

**Affiliations:** 1 Malopolska Centre of Biotechnology, Jagiellonian University, 30-387 Kraków, Poland; ewelina.pospiech@uj.edu.pl; 2 Institute of Computer Science, Polish Academy of Sciences, 01-248 Warsaw, Poland; Pawel.Teisseyre@ipipan.waw.pl (P.T.); jan.mielniczuk@ipipan.waw.pl (J.M.); 3 Faculty of Mathematics and Information Science, Warsaw University of Technology, 00-662 Warsaw, Poland; 4 Central Forensic Laboratory of the Police, 00-583 Warsaw, Poland

**Keywords:** physical appearance, human genome variation, DNA-based prediction, investigative leads, forensic DNA intelligence, forensic genomics

## Abstract

The idea of forensic DNA intelligence is to extract from genomic data any information that can help guide the investigation. The clues to the externally visible phenotype are of particular practical importance. The high heritability of the physical phenotype suggests that genetic data can be easily predicted, but this has only become possible with less polygenic traits. The forensic community has developed DNA-based predictive tools by employing a limited number of the most important markers analysed with targeted massive parallel sequencing. The complexity of the genetics of many other appearance phenotypes requires big data coupled with sophisticated machine learning methods to develop accurate genomic predictors. A significant challenge in developing universal genomic predictive methods will be the collection of sufficiently large data sets. These should be created using whole-genome sequencing technology to enable the identification of rare DNA variants implicated in phenotype determination. It is worth noting that the correctness of the forensic sketch generated from the DNA data depends on the inclusion of an age factor. This, however, can be predicted by analysing epigenetic data. An important limitation preventing whole-genome approaches from being commonly used in forensics is the slow progress in the development and implementation of high-throughput, low DNA input sequencing technologies. The example of palaeoanthropology suggests that such methods may possibly be developed in forensics.

## 1. Introduction

**Figure 1:**
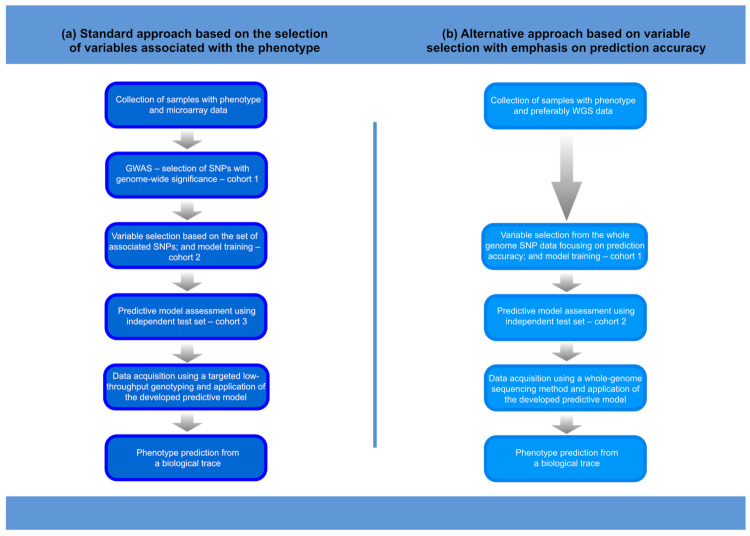
Procedure for the development and application of a phenotype prediction tool. The main differences in the procedures for developing a predictive model using the standard or alternative approach concern the selection of variables and the number of variables in the model. Consequently, the method of acquiring genetic data in the practical forensic applications of the next-generation predictive models may require whole-genome sequencing methods. Thus: (**a**) only phenotype-associated SNPs are included in prediction modelling, the models are not very extensive, and the methods of data acquisition can be less complex (SNaPshot, targeted MPS); (**b**) the selection of relevant variables (SNPs) is targeted towards improving the prediction accuracy of the model, and much more advanced variable selection methods are required. Some complex models may involve many thousands of SNPs, which, in biological traces, must be analysed using whole-genome sequencing methods that are effective for low DNA input samples.

The information included in genomic data can be used to generate investigative leads that, when properly used, can speed up the process of human identification in forensic investigations. Such forensic DNA intelligence can use a variety of methods, including relatedness testing, the inference of ancestry, the prediction of physical phenotype, and age estimation [1,2,3,4]. As an inherently interdisciplinary science, forensic science today can benefit from the rapidly developing methods in the areas of genomics and machine learning, which is particularly beneficial for the further development of forensic DNA intelligence. Studies of human genome variation conducted today on an unprecedented scale are revealing how genes control phenotypes. This knowledge has fundamental meaning for understanding the genome–phenome relationship. Importantly, the growing knowledge of human genome variation allows for the development of algorithms that can more accurately predict phenotypes, providing more reliable investigative leads to help identify an unnamed perpetrator or victim and solve a case. It is worth noting that the DNA-based predictive tools developed in the forensic field are also useful in evolutionary anthropology. In this review paper, we will summarise how the advances in understanding the genetic architectures of various human physical characteristics, and the progress in high-throughput genotyping technologies in combination with machine-learning methods, allow the prediction of physical appearance traits. We will also highlight the evolution of the approach to the genetic prediction of physical traits, which has moved from building predictive models based on variables that show genetic association to building models based on variables that improve predictive performance (Figure 1).

## 2. Explaining the Heritability of Appearance Traits

A large meta-analysis of twin studies has confirmed that all human traits are heritable and showed that most of the traits can be explained by an additive genetic variation [5]. The extreme similarity of physical appearance of monozygotic twins clearly indicates the role of genes, but their identification is not simple due to the complex nature of appearance traits. Linkage mapping, which relies on the co-segregation of causal DNA variants with marker variants (SNP or STR) within pedigrees, has been very successful at identifying the gene variants affecting simple mendelian traits [6], but mostly failed to identify the DNA variants involved in the determination of complex traits [7]. The breakthrough in explaining the heritability of complex phenotypes has come with the advent of genome-wide association studies (GWAS), which are effective at discovering common variants with small effect sizes on traits [7]. GWAS is used to identify associations between genotypes and phenotypes by testing for differences in the allele frequency of DNA variants between individuals who differ phenotypically. Technically, the analysis of hundreds of thousands of DNA variants in the genomes of these individuals enables finding those statistically associated with a specific phenotype [8].

### 2.1. Pigmentation Phenotype

GWAS data have been very effective at explaining the heritability of physical appearance traits. The heritability of human pigmentation traits has been assessed to be above 80% and, thus, provides a good starting point for DNA-based prediction, because it means that 80% of the variation in pigmentation in a population is due to genetic variation between individuals and that the influence of the environment is relatively small [9,10,11].

Many candidate genes for human pigmentation were identified before the GWAS era through animal models and the linkage to diseases with mendelian inheritance modes, such as oculocutaneous albinism. Genome-wide association scans confirmed the importance of these genes and identified many of the novel gene variants influencing the variability of normal human pigmentation. The collected data confirmed a very promising perspective for the genetic prediction of pigmentation traits. The less complex nature of some pigmentation phenotypes, such as blue and brown eye colours and red hair colour, and the availability of DNA variants with relatively large effect sizes, similar to the genetic effects observed for mendelian traits, were particularly encouraging. The region on chromosome 15, including the *OCA2* gene, was implicated in eye colour via linkage and subsequent fine-mapping analyses [12,13]. The evidence of a relationship between *OCA2* genotypes and eye colour became stronger with additional reports [14,15,16]. This was an Icelandic GWAS that implicated the involvement of neighbouring *HERC2* in the determination of eye colour and suggested that this genomic region was responsible for the regulation of *OCA2* gene expression [17]. This speculation was soon confirmed by other studies that showed that the DNA variant rs12913832 was responsible for brown and blue eye colour in humans [18,19]. The postulated interaction between these two genes in determining eye colour was also confirmed [20]. Most of the SNP heritability of red hair colour is explained by the single *MC1R* gene, which was also discovered long before the genome-wide association scans and confirmed in various population samples across the globe [21,22]. The effect of this gene was extended to skin colour and freckling [23,24]. These early genome-wide association scans for pigmentation also clearly demonstrated their agnostic power to detect novel, sometimes unexpected genotype–phenotype relationships, as in the case of the *IRF4* gene, which is now an important predictor of pigmentation phenotypes [17,25]. The success of GWAS was clear, but a significant proportion of heritability remained missing, which could be attributed mainly to the insufficiently large sample sizes used in genome-wide association scans, insufficient phenotyping regimes generating heterogeneity, the insufficient density of the GWA arrays and the significance of non-additive variation [26,27]. Indeed, the improved statistical power to detect small effect-size variants more effectively in the next series of genome-wide association scans enabled the identification of multiple new DNA variants involved in the heritability of hair and eye colour. For example, a large study of 192,986 European individuals from 10 populations identified 50 new loci for eye colour [28]. The study revealed signals with genome-wide significance for 12,192 SNPs from 52 genomic regions in the discovery set of 157,485 individuals. By combining discovery and replication sets, the study finally identified 124 independent associations from 61 genomic regions and concluded that the known variants explain 53.2% of eye colour variation. Notably, the study also investigated Asian cohorts and found consistency in the genetic architecture of eye colour in populations from Europe and Asia [28].

Human skin colour is highly variable at continental and intercontinental levels, complicating research on the genetic architecture and heritability of this trait [29]. The rs1426654 in *SLC24A5*, discovered thanks to a Zebrafish study, plays an important role in skin colour differences at the continental level, explaining more than 30% of skin colour differences between African and European populations [30]. GWAS on skin colour conducted on various population samples discovered multiple genes and gene variants involved in skin colour variation at the intercontinental level [25,31,32,33,34]. Notably, the studies of African populations showed large differences in skin colour, revealing the high complexity of the genetic architecture of skin colour in Africa and the significance of genes unknown to European studies [35,36].

A meta-analysis that involved almost 300,000 genomes from individuals of European ancestry included in two different cohorts (23andMe, UK Biobank) discovered 124 loci relevant to human hair colour, mostly novel associations, including genes with strong effect, such as *SLC45A1*, *DSTYK*, *FOSL2*, *LHX2*, *EDNRB*, *SHC4*, *KRT31*, and *BCAS1*. The study was highly successful at explaining up to 34.6% (red hair) of the heritability of human hair colour, despite an imperfect phenotyping regime involving self-reported hair colour in adulthood [37]. Another study based on UK Biobank resources examined 343,234 genomes from participants reporting British descent and these were, thus, more homogeneous. This study assessed that all identified variants explain 90% of the SNP heritability of red hair colour but, surprisingly, it found that a DNA variant located 97 kb from the 5’ end of the *MC1R* gene may be more important for explaining red hair colour than the polymorphism within the *MC1R* exon, and it identified an additional eight loci that contribute to the genetics of red hair colour. This research also revealed 213 variants important to the determination of blond hair colour, accounting for 73% of SNP heritability. In addition, a set of 56 DNA variants was found to be important for brown hair colour and was assessed to account for 47% of SNP heritability of this hair category [38].

### 2.2. Hair Features

Along with hair colour, other features describing the properties of human hair can be useful to define the physical appearance of an individual. Research shows that genetics plays a key role in the determination of hair features. However, the level of heritability may differ for various hair traits. Very high heritability (85–95%) was estimated for hair shape [39]. Heritability of around 70% was reported for monobrow and beard thickness [40]. Studies are contradictory in terms of the heritability level of hair loss (~40–80%) [40,41,42,43] and hair greying (~30–90%) [40,44], but the accuracy of the heritability measurement may be affected by the definition of heritability, the study design and the method of analysis used [45]. It is worth noting that heritability values calculated from the entire SNPs analysed in GWA studies tend to be underestimated compared to estimates of pedigree heritability, because the former do not include phenotypic variation due to rare variants that are not correctly determined by the SNPs genotyped on microarrays or common variants with small effect sizes that are not correctly identified if the sample size is not large enough. In turn, pedigree heritability may be biased by the common environmental factors to which families are typically exposed [43,45]. Notably, heritability estimates may vary due to changes in allele frequencies in populations caused by different evolutionary mechanisms and environmental contributions that change with the age of individuals [46]. The genetic basis of hair loss and hair shape are the most investigated so far. Androgenetic alopecia, known in men as male pattern baldness (MPB), is the most common type of progressive loss of hair from the scalp and is particularly frequent among men in Europe. Over the last >10 years, several GWA studies on hair loss have been carried out, with the vast majority of research conducted on Europeans [43,47,48,49,50,51,52,53]. These studies revealed multiple genes that are associated with the risk of MPB, with two loci showing the strongest effect of association, Xq12 (*AR*/*EDA2R*) and 20p11 (*PAX1*/*FOXA2*). Of these two loci, only 20p11 is known to act in Asians, indicating the significance of population heterogeneity [54,55]. A long list of additional loci, representing smaller effect sizes, identified through GWAS and/or candidate gene approaches, is available in the literature (e.g., *HDAC9*, *WNT10*, *TARDBP*, *EBF1*, *SUCNR1*, *AUTS2*, *FGF5*, *IRF4*, *C1orf127*, *RUNX1,* and *TWIST2*). Studies published in 2017–2018 led to significant advances in research on the genetics of hair loss. Four large GWA scans have been conducted on individuals of European descent. The first three of those studies, which investigated 20,000–50,000 genomes each, detected altogether more than 300 GWA signals, including 253 novel MPB associations [42,52,53]. The largest study, which investigated 200,000 genomes, allowed the identification of >600 genome-wide associations, explaining altogether 25% of the phenotypic variation observed in alopecia [43]. These large-scale studies not only discovered many new loci involved in alopecia, but also highlighted the implicated molecular pathways and discovered the genetic links of alopecia with different traits/conditions, including bone mineral density, puberty, metabolic traits, and Parkinson’s disease. However, a significant part of MPB heritability remains missing. Recent studies showed that the use of advanced statistical methods and the incorporation of functional genomics data prior to association tests may improve the efficiency of SNP detection in GWAS and these approaches were proved to be successful in MPB research by increasing the number of SNP hits by an additional ~4% [56,57].

For hair morphology (shape), four GWA studies have been published so far, two of which were carried out on Europeans, with one study on Latin Americans and one on East Asians [40,58,59,60]. Hair shape, usually defined as straight vs. wavy vs. curly, is a highly distinctive feature of human appearance. As with hair loss, genetic heterogeneity between populations is observed with different mechanisms and genes underlying straight hair formation in Europeans and East Asians. The *TCHH* gene is known to act only in Europeans, while *EDAR* is the main contributor to straight hair in East Asians [58,59,61]. However, the proportion of known heritability attributed to both genes in respective populations was found to be small (<10%). The *TCHH* gene was discovered in the first GWA study published in 2009, which was conducted on three cohorts with a total of more than 4800 individuals of European descent [58]. *TCHH* was the only gene in this study that reached genome-wide significance, but suggestive associations were also disclosed for several additional loci, including the *FRAS1* and *WNT10* genes. The role of the *EDAR* gene in hair straightness and thickness in Asians was discovered through candidate gene analyses [61,62] and confirmed in a later GWA study conducted in 2016 on ~2900 Chinese people, with no additional genes reaching GWA significance in this study [59]. A GWA study conducted on >6000 Latin Americans discovered a novel association for *PRSS53* [40]. The latest meta-analysis of European GWA studies, exploring a total of more than 16,000 samples, allowed the identification of 12 hair shape genes, including eight novel association signals (*ERRFI1/SLC45A1*, *PEX14*, *PADI3*, *TGFA*, *LGR4*, *HOXC13*, *KRTAP*, and *PTK6*) [60]. The study showed that a model consisting of 14 SNPs across novel and literature loci, together with sex, explains 10% of the total hair shape variability. Further research pointed to the role of gene–gene interactions in hair shape determination as one of the factors underlying missing heritability [63]. The *RPTN* gene has been implicated in straight hair formation in Europeans and East Asians, but throughout interactions with different previously known head hair shape genes.

Only a few studies have addressed the genetic basis of other hair traits. In recent years, the first genes responsible for the thickness of the eyebrows, e.g., *EDAR*, *FOXL2*, *LIMS1*, *TMEM174*, SOX2, and *FOXD1* [40,64,65], monobrows, e.g., *PAX3* and 5q13.2 [40,51], beard thickness, e.g., *EDAR*, *LNX1*, *PREP*, and *FOXP2* [40], and hair greying, e.g., *IRF4*, and *KIF1A* [40,66], have been identified through whole-genome or whole-exome analyses.

### 2.3. Human Height

Human height is heritable in approximately 80%, but only the single genes associated with this trait were known before the era of GWA studies. The study of human height genetics is a good example of the effectiveness of explaining the heritability of complex traits through the GWAS approach. An important advantage of stature studies is undoubtedly the ease of measuring this phenotype and thus the homogeneity of the phenotypic data. The genome-wide association scans for human stature identified gene variants with a small effect size, clearly confirming the high polygenicity of this trait. The first three GWA studies of human height, which collectively included more than 50,000 samples, detected only 54 loci with a statistically significant association with stature [67,68,69]. Most of these loci have not been previously linked to human height and, in many cases, the known biological function did not make them candidates for the regulation of human stature. The genes discovered explained only about 5% of the variation in height, which was very discouraging, especially for predicting this distinctive feature. A huge meta-analysis that considered GWAS data for more than 180,000 genomes made only a small advance in explaining the heritability of human growth, enabling the discovery of 180 loci. The work demonstrated the importance of allelic heterogeneity in explaining the complex genetic architecture of human stature [70]. At the same time, it has been argued that testing each SNP individually for an association with a trait, which is typical for GWAS investigations, leads to missing many real associations, especially when the effect sizes of individual SNPs on a trait are small. By fitting all SNPs simultaneously, Yang et al. provided an unbiased estimate of the variance explained by the SNPs in total, and showed that common genetic variants are able to explain as much as 45% of the variance in human height [71]. However, consistently increasing the number of genomes analysed with high-density DNA microarrays has proven to be an effective method for elucidating the still-missing genetic variation responsible for human height. The large meta-analysis that included 700,000 European genomes (250,000 previously investigated [72] and 450,000 from the UK Biobank) identified 3290 near-independent SNPs associated with human stature which were found to explain 24.6% of variance of this trait [73]. Still, unpublished data suggest that the large proportion of missing heritability may be hiding in rare genetic variants (≤0.01) that can be detected via the whole genome sequencing of a sufficient number of genomes [74]. Notably, Zoledziewska et al. showed that human height can be under pressure from natural selection, presenting data showing that known height-decreasing alleles were found at higher frequency in Sardinians than would be expected to be caused by genetic drift [75]. Research on the genetic architecture of human stature, on a smaller scale, is also being conducted in populations in Asia and Africa. A meta-analysis of 93,926 individuals from East Asia identified 98 loci, including 17 novel for human height [76]. A GWA study based on 191,787 Japanese genomes disclosed 573 height-associated variants and assessed that 64 rare (<0.01) and low-frequency (<0.05) variants explain 1.7% of the height variance. The study revealed genes not previously associated with stature [77]. Eighty-three low-frequency variants affecting human height have also been reported in [78].

### 2.4. Facial Morphology

The human face represents a set of correlated complex phenotypes that are highly variable at inter- and intra-population levels and define what is apparently the most differentiating human trait [79]. The high similarity of the faces of monozygotic twins clearly indicates that most of this variability is genetically determined. Despite this, research into the heritability of facial features has caused quite a few problems, probably due to the three-dimensional nature of human faces. Only a recent face heritability study performed on 952 British twins using an advanced phenotyping and landmarking system confirmed the high heritability (>65%) of many facial traits [80]. Indeed, contrary to some physical phenotype traits, collecting phenotypic data for faces can be challenging. A self-reported categorisation is less useful, and measurement ideally requires the involvement of methods that are able to capture the three-dimensionality of faces. The approaches used to collect facial appearance data for studying the genetics of craniofacial variation that can be found in the literature are standard 2D photographs, magnetic resonance imaging (MRI) and 3D scanning. The latter has quickly gained a dominant position in craniofacial genetics research. It should be noted that the phenotypic assessment of facial variability from 3D images is not an easy task and makes large-scale studies and comparisons between different studies difficult. Initially, the process relied on a labour-intensive process of the manual determination of landmarks, and later, several automated landmarking methods applicable to 3D images have facilitated research on the association of facial phenotype with genotype [80,81,82,83]. In one of the first works on the genetics of natural craniofacial variation, 11 DNA variants previously associated with cleft-lip phenotypes were tested in two European cohorts with the phenotypes captured using 2D photos or magnetic resonance images [84]. A DNA variant near the *GREM1* gene was associated with nose width, and another near the *CCDC26* gene was associated with bizygomatic distance. The first GWAS scan that was aimed at investigating normal facial variation identified only a single intronic DNA variant in the *PAX3* gene, which showed association with nasion position. This first study was conducted on a relatively small group of 2185 adolescents. The study used a 3D laser scanning method to collect phenotypic data. The 22 identified landmarks were then used to generate 54 3D and 2D distances featuring different facial characteristics. Additionally, following a previous method, a principal component analysis enabled the identification of 14 independent groups of correlated coordinates [85]. These parameters were used in association testing, which identified four associations, but only *PAX3* was replicated in an independent cohort of 1622 participants [86]. A larger GWAS analysis of almost 10,000 individuals of European origin from several cohorts used 3D MRI scans and 2D photos, and identified five genes involved in facial variation. *PAX3*, *PRDM16*, and *TP63* have previously been linked to craniofacial development, while *C5orf50* and *COL17A1* were new findings [87]. The strongest signal was again obtained for *PAX3*, which soon gained further confirmation in an independent study of about 6000 Latin Americans investigated in the large CANDELA project [40]. It is worth noting that rare variants in *PAX3*, the most replicated gene for natural variation in facial appearance, cause Waardenburg syndrome, which involves some facial dysmorphism, including a broad nasal bridge. The phenotyping regime in Adhikari’s study involved a simple approach based on standard 2D photographs, and the study also implicated *DCHS2*, *RUNX2*, *GLI3*, and *PAX1* in nose morphology and *EDAR* in chin protrusion [40]. Another GWAS study, which included 3D images of 3118 individuals of European ancestry that were used to derive 20 facial distance measurements, identified several genomic regions and implicated *MAFB*, *PAX9*, *MIPOL1*, *ALX3*, *HDAC8*, and *PAX1* in normal facial variation, including the measures of eye, nose, and facial breadth. The study also provided additional evidence for the association between *PRDM16* and *C5orf50* and facial features [88]. Crouch et al. investigated the hypothesis that the DNA variants responsible for large effects on facial morphology exist in the human genome, and focused on individuals displaying extreme facial characteristics to find them. The study included 3D images of 1832 individuals from the general population as a discovery set and 1567 3D scans of twins from the TwinsUK databank, plus 33 of East Asians for replication. The original 3D scans were used to manually mark each face with 14 well-defined landmarks, allowing a mesh of 50,000–150,000 surface points in 3D space to be transformed into a set of 29,658 surface points for each face. This approach enabled the identification of three SNPs in *PCDH15*, *MBTPS1*, and *TMEM163*, genes that have previously been associated with various pathological phenotypes involving craniofacial dysmorphias [89]. The study by Claes et al. (2018) involved 2329 individuals at the discovery stage and an additional 1719 at the replication stage, and found associations for 15 loci with facial features, including four new genes, nine consistently confirmed, and two linked with pleiotropic facial phenotypic features. The study used an innovative, data-driven facial phenotyping approach based on structural correlations between about 10,000 3D quasi-landmarks, which enabled the hierarchical (global-to-local) clustering of the human face into segments [90]. This approach also yielded good results for a meta-analysis, which included 8246 European individuals and enabled the identification of 203 loci associated with normal facial variation [91] and for a study of facial features in East Africans, which investigated 2595 3D facial images collected on Tanzanian children [92]. The latter cohort was previously investigated, with two genes, *SCHIP1* and *PDE8A*, identified that were associated with measures of human facial size [83]. GWA studies investigating human facial morphology in non-European cohorts are rare. Worth noting is a GWAS conducted on an exploratory panel of Uyghurs that identified six loci important for the genetic architecture of the human face, four of which were replicated in independent cohorts of Uyghur or southern Han Chinese [93].

## 3. DNA-Based Predictive Tools for Forensic Applications

Several factors determine the accuracy of DNA-based predictive methods, including high heritability of a trait, the identification of appropriate predictors, and the selection of the best mathematical approach to model development. The forensic community very early recognised the investigative potential of extracting phenotypes from DNA data. The practical importance of a simple amelogenin genetic sex test [94], and also of the inference of biogeographical ancestry [95,96], made it clear that a description of the phenotypic characteristics of a person of undetermined identity can provide important investigative leads. The variation of the *MC1R* gene was soon proposed as an indicator of red hair colour [97], while the predictive potential of the *OCA2* variation was proposed for the inference of eye colour [14]. The availability of GWAS data has made it possible to develop tools for predicting human appearance traits more effectively. The research carried out has made it possible to develop predictive tools with varying performances and practicalities of application for different physical characteristics (Table 1).

### 3.1. Pigmentation Traits

In particular, the discovery of eye colour markers with large phenotypic effects has made it easy to develop pretty accurate genetic predictors of this trait. The best-known tool commonly used in the forensic field today is the IrisPlex predictive system, which includes both a genetic test for data acquisition and a mathematical algorithm for predicting the three categories of eye colour [98]. The algorithm was developed based on the systematic selection of markers made by Liu et al., who reported 24 variants from eight genes, enabling the prediction of blue and brown eye colour with a prediction accuracy expressed by an AUC of 0.91 and 0.93, respectively [122]. AUC, which stands for area under the ROC (receiver operating characteristic) curve, describes the general performance of the model in such a way that 1 means perfect classification and 0.5 means random assignment to the phenotype categories. For forensic purposes, the number of markers from the originally identified 24 was restricted to the six with the largest effect [98,122]. The six crucial predictors included *HERC2* rs12913832, *OCA2* rs1800407, *SLC24A4* rs12896399, *SLC45A2* rs16891982, *TYR* rs1393350, and *IRF4* rs12203592. The original IrisPlex method implements a multinomial logistic regression algorithm and a simple single base extension method based on SNaPshot minisequencing, which allows the PCR amplification and genotyping of several SNPs in a multiplex reaction. Importantly, the products of primer extension are analysed using capillary electrophoresis platforms, which are commonly used in human identification testing laboratories. Other tools based on other mathematical solutions were soon developed but, essentially, each of these algorithms relied on exploiting information in the *HERC2-OCA2* gene complex. In general, these works were limited to the development of predictive algorithms using various sets of samples and mathematical approaches, but did not present specific tools for the collection of genetic data [99,100,101,102,103,104]. Notably, IrisPlex and other forensic methods of eye colour prediction can accurately predict blue and brown iris colours, but have difficulty with the prediction of intermediate eye colours [3]. Moreover, in some populations, the effect of sex was noted on prediction results [123,124,125]. The IrisPlex tool for the genotyping and prediction of eye colour evolved to HIrisPlex [106] and finally to the HIrisPlex-S tool [109], which were developed based on the same strategy as IrisPlex. The algorithm for hair colour prediction implemented in HIrisPlex was developed based on the investigation of a Polish population sample, which enabled the selection of 22 crucial SNPs from 11 genes for hair colour. The study showed a high level of accuracy for red and black hair colour prediction (AUC ~ 0.9) and a lower prediction accuracy for blond and brown hair colour (AUC ~ 0.8) [126]. The skin colour predictor was proposed by Walsh et al. after a systematic study of skin colour candidate variants in a sample of 2025 individuals from 31 worldwide populations. The algorithm predicted skin colour with very high accuracy, with an AUC = 0.97 for light skin colour, 0.83 dark, and 0.96 for dark-black skin colour [127]. Notably, it has been demonstrated that the original SNaPshot protocol can be replaced by the targeted massive parallel sequencing (MPS) method [128], and the HIrisPlex-S method was also adopted in a tool combining pigmentation prediction capability with ancestry inference developed by the VISAGE consortium [129]. Other studies also investigated the possibility of hair and skin colour prediction in the forensic field [100,105,107,108,110]. The Snipper Application suite deserves more attention because it provides an online tool that allows the performance of predictive calculations based on data generated by any genotyping method. The tool was originally developed for the statistical interpretation of data in ancestry inference studies, but a number of new functionalities have subsequently been added to enable the prediction of pigmentation and even age [130]. A more complete prediction of pigmentation will be provided by the developed algorithms for freckle prediction [111,112]. It is worth noting that the use of extended DNA variant sets for prediction has begun to be explored, which may lead to the development of next-generation prediction tools. For example, the previously described association work of Hysi et al. was extended to predictive modelling. Hair colour prediction was compared in two independent cohorts using prediction models based on the 258 associated SNPs and the original HIrisPlex method, and these new models outperformed the previous HIrisPlex model [37]. Further development of pigmentation predictors may also require the use of sex information, and age will naturally be needed for the final interpretation of the data [37,123]. This issue is also addressed later in the article, as sex in particular can be important for predicting other appearance traits.

### 3.2. Hair Loss

Numerous association studies conducted for MPB raised questions about the predictive ability of the discovered genetic variants. In 2015, a compact regression model was developed based on analysis of five SNPs from five genomic regions (Xq12, 20p11, *EBF1*, *TARDBP*, and *HDAC9*), trained and validated on >600 samples from six European populations [113]. The model was shown to enable the prediction of hair loss in Europeans at an acceptable level, but only in two extreme phenotype categories, i.e., young men with significant alopecia vs. older men without symptoms of alopecia with AUC of 0.76. In the same study, Marcińska et al. also pointed to the potential role of allelic heterogeneity in determining scalp hair loss. Expanding the number of DNA variants in both crucial regions, i.e., Xq12 and 20p11, improved the accuracy of prediction, suggesting that there might be more functional variants in these loci. The extended 20-SNP regression model predicted hair loss with an AUC of 0.66 in all samples of all age categories and had the highest AUC value for the age category of ≥50 years old (AUC = 0.76; sensitivity = 67.7%; specificity = 90%), where the sensitivity refers to the ability of the model to correctly classify individuals with the particular phenotype (here baldness), while the specificity refers to the ability of the model to correctly classify individuals without this phenotype [113].

Liu et al. conducted a parallel study on the prediction of MPB in >2700 Europeans and developed a 14-SNP model that was found to predict early-onset MPB cases with a cross-validated AUC of 0.74 [114]. The accuracy of hair loss prediction status in elderly and middle-aged individuals was lower, with an AUC of 0.69–0.71. In 2017, Hagenaars and colleagues developed a polygenic predictor based on the genome-wide data generated for a large cohort of 40,000 individuals and showed that it can discriminate individuals with no signs of hair loss from those with severe baldness, with an AUC = 0.78, sensitivity = 0.74, and specificity = 0.69 [52].

### 3.3. Hair Shape and Other Hair Features

The first preliminary model for head hair shape was developed as a follow-up to the first GWA study conducted on hair characteristics [58], and included an analysis of three SNPs in three genes (*TCHH*, *WNT10A*, *FRAS1*), and was trained on data generated for 528 samples from Polish individuals [115]. The model was reported to predict straight hair with high accuracy but low specificity (cross-validated AUC = 0.622, sensitivity = 93.2%, specificity = 15.4%). The application of the model on an independent test set consisting of samples from six European populations and using a 65% probability threshold allowed for higher sensitivity (81.4%) and improved specificity (50.0%) of prediction, but at the same time with a very high rate of inconclusive results (66.9%). In 2018, a large-scale prediction study for hair shape prediction was conducted with more than 9600 samples used for predictor selection and model development and more than 2400 samples used for prediction model validation, collected from both European and non-European populations [116]. The binomial logistic regression model was developed to predict hair shape, defined as straight vs. non-straight, based on 32 informative SNPs from 26 loci. The model was reported to explain ~12% of hair shape variation and can predict straight vs. non-straight hair in European populations with an accuracy of AUC of 0.66, a sensitivity of 84.1% and a specificity of 34.2%. It was shown that the same set of SNP markers can predict hair shape with significantly different accuracies in Europeans and non-Europeans. For non-European samples, the AUC value was 0.79, sensitivity = 82.9%, and specificity = 49.8%. The higher prediction accuracy obtained for non-European populations compared to Europeans is due to the effect of the *EDAR* gene, which has a significant effect on the determination of straight hair in non-European populations, primarily East Asian. In addition to the binomial model, a multinomial logistic regression model was developed to allow for a higher resolution of hair shape prediction, considering three categories—straight, wavy and curly—based on an analysis of 33 SNP positions. There are few or no prediction studies of the remaining hair features. In 2016, Adhikari et al. predicted different hair traits using the GWAS data generated for Latin Americans and reported the highest accuracy of prediction for beard thickness and the lowest for hair greying, with ~18% and ~7% of the phenotypic variation explained by the associated SNPs, respectively [40]. Interestingly, for both of these traits, a large effect of age and sex on prediction was observed, explaining the additional ~11% and ~20% of the phenotypic variation, respectively, for beard thickness and greying. Age was found to be a main predictor of hair greying in a study conducted in 2020, explaining around 48% of the variation observed in hair greying in a cohort of 849 people from Poland [66]. A binary neural network model for greying vs. no greying prediction was developed in this study based on information relating to age, sex, and 10 SNPs selected using whole-exome sequencing data analysis (e.g., *KIF1A* rs59733750, *SEMA4D* rs45483393) and literature resources (*IRF4* rs12203592, *FGF5* rs7680591). The model achieved a high accuracy of prediction with a cross-validated AUC = 0.87 (sensitivity = 0.73; specificity = 0.85) but most of the prediction information was driven by age itself, while SNPs were found to explain merely ~7% of the variation in hair greying. As mentioned earlier, age is a very important factor in predicting hair loss. Sex and age were also shown to slightly improve the accuracy of prediction of hair shape [116].

This implies that there is a need to determine the sex and age of an individual from the analysed biological sample. Information on a person’s sex is usually available in criminal investigations due to the inclusion of marker for the amelogenin gene located on the X and Y chromosome in standard STR DNA profiling, as previously mentioned, whereas age can be estimated via epigenetic analysis [131].

### 3.4. Human Stature

Attempts at forensic human height prediction have not been particularly numerous and have been limited to the development of predictive algorithms that are not equipped with data collection tools. The reasons are related to the limitations of DNA analysis technology and stem from the need to analyse too many DNA variants. While the 5% heritability explained by the 54 DNA variants identified by the initial GWAS scans for human height was unlikely to predict the full range of human height, Aulchenko et al. tested whether it would allow the reliable prediction of extreme height. However, this turned out to be possible with only limited accuracy. Tall stature prediction was possible at AUC of 0.65, thus only moderately improving the accuracy resulting from a random hit (AUC = 0.5) [117]. Using the 180 height markers identified in the Lango Allen et al. paper improved the prediction of tall stature to AUC of 0.75 [118]. The study suggested the importance of allelic heterogeneity for the prediction of human stature. Further increasing the number of predictors to 697 reported in the paper by [72] enabled the prediction of tall stature with an AUC of 0.79 [119]. The possibilities of human height prediction have also been explored outside the forensic mainstream using a non-standard approach that has nevertheless yielded very promising results, enabling the prediction of the full range of human height at a good level of accuracy [120]. Based on the results obtained, the authors suggested changing the approach to phenotype prediction, pointing out the benefits of also including as predictors polymorphisms that do not show an association with a given trait, but only on the basis of the improved prediction accuracy obtained after their inclusion in the prediction model [132].

### 3.5. The Human Face

Drawing a forensic sketch based on the instructions of a witness in a criminal case is a tool that has been used for years to identify the perpetrator of a crime. People recognise each other through the high variability of facial features. Therefore, having a good understanding of the genetics of human facial variation and being able to predict this complex phenotype is a very exciting prospect for forensic DNA intelligence. The small amount of explained heritability for craniofacial traits does not bring good prospects for the prediction of human facial phenotypes. Nevertheless, attempts have been made to develop models that would allow the prediction of facial appearance. The proposed methods are based on the indirect prediction of facial phenotypes, with ancestry and sex prediction DNA data playing a key role in this regard. The method by Claes et al. implements a bootstrapped response-based imputation modelling that makes use of information on genomic ancestry and sex first to create a sketch called a base-face. At the second stage, the information in 24 SNPs associated with facial variation is used to improve the prediction outcome [82]. A similar strategy was proposed by Lippert et al., who used the whole genome sequencing data to gain information about the sex and ancestry proportions of the individual [121]. The data on genetic face predictors did not improve facial appearance predictions, but the study showed a positive effect on the prediction of age and body mass index. The genetic prediction of facial features was also explored by Qiao at el., who developed a quantitative model based on multiple SNP loci and tried to simulate 3D face models. The study suggests that epistasis is part of the genetic architecture of facial features and concludes that the model developed should be treated as an exploratory basis for future, more advanced predictive models [93].

## 4. Appearance Prediction in the Era of Big Data

### 4.1. Appearance Trait Predicition as a Supervised Learning Task

The prediction of human externally visible characteristics using DNA markers can be treated as a supervised learning problem in which the considered appearance trait corresponds to a response (target) variable, whereas genetic markers correspond to explanatory variables (also known as features or predictors). The supervised learning models are fitted using training data, which consist of observations for which the value of the target variable is known. Depending on the type of the target variable, three tasks can be distinguished: regression (for a quantitative trait, e.g., human height), binary classification (for a binary trait, e.g., the presence of freckles), and multi-class classification (for a categorical trait, e.g., eye colour).

The specificity of the problem and the greatest challenge lies in the large number of potential features (genetic markers), which may significantly exceed the number of observations in the training data. Due to this, the use of traditional models and estimation methods (such as the maximum likelihood method in logistic regression) is not feasible. The simplest solution is to use some initial filtering method to reduce the total number of markers. However, simple filters only assess the marginal dependence between the variable and the trait; they may exclude variables that are potentially useful for the model, for example, variables that contribute by interacting with already selected ones. Therefore, there is a need to apply the estimation methods as well as feature selection approaches specially tailored to high-dimensional settings. This is one of the greatest challenges in designing learning models for appearance trait prediction.

Finally, it is important to note that traditional genome-wide association studies focus on detecting the genetic variants associated with the trait with high statistical confidence, which, in particular, includes controlling the probability of at least one rejection via multiple-testing procedures. When the prediction is the main task, the paradigm shift is needed, because focusing on the accuracy of the model becomes the main objective [133]. This approach requires the careful selection of variables. On one hand, unlike in GWAS, it is allowed to include a certain number of non-significant variables in the model, since the excessive pruning of SNPs, which may result in the discarding of some significant variables, can negatively affect prediction accuracy [132]. On the other hand, including too many spurious variables may cause the overfitting of the model and decrease its accuracy [134].

### 4.2. Linear Easily Interpretable Models

Despite its simplicity, the linear model and its generalisations are powerful tools for appearance trait prediction. The theory [135] and empirical evidence [136,137] suggest that in many cases the dependence between the trait and genetic markers can be captured using linear models. Several studies indicate that they frequently work on par or even better than more complex models, such as ensemble methods or neural networks [120,132,135,136,137], as they are not liable to overfitting. A distinct advantage of the linear models is their interpretability; the parameter value indicates how the given variable influences the dependent variable for fixed values of the remaining variables. Within the linear models, there are many methods of parameter estimation, among which the regularised (also known as penalised) maximum likelihood methods play the most prominent role in modern genetic data analysis. First, for the regularisation methods, there are theoretical guarantees that the solution of the related optimisation problem exists and is unique, even for a high-dimensional setting. Second, some forms of the regularisation, such as lasso, ensure the sparsity of the vector of estimated coefficients, meaning that a majority of coefficients will be zero. Under some unfortunately stringent conditions, this majority will correspond to non-significant variables in the model. Thus, the selected regularisation techniques can be seen as methods of simultaneous parameter estimation and feature selection. Below, we discuss the three most important generalised linear models (linear regression, logistic regression, and multinomial regression) and the methods of parameter estimation within them.

In the case of the quantitative trait, it is natural to consider the *linear regression model*, which assumes that for an i-th observation, we have $y_{i}=\beta_{0}+x_{i}^{T}\beta +\epsilon_{i},$ where $y_{i}$ is the value of the target variable, $\beta_{0}$ is an intercept, $\beta ={\left(\beta_{1},\ldots ,\beta_{p}\right)}^{T}$ is the coefficients vector, $\epsilon_{i}$ is noise, and $x_{i}={\left(x_{i,1},\ldots ,x_{i,p}\right)}^{T}$ is a vector of features. Coordinates $x_{i,1},\ldots ,x_{i,p}$ denote the genetic markers for the i-th observation. They can be coded numerically as 0, 1, or 2, where 0 indicates the homozygosity of the major allele, 1 the heterozygosity and 2 the homozygosity of the minor allele. In the penalised least squares method, we solve:

$${\hat{\beta }}_{0},\hat{\beta }=\arg\underset{b_{0}\in R,b\in R^{p}}{\min}\overset{n}{\underset{i=1}{\sum }}{\left(y_{i}-b_{0}-x_{i}^{T}b\right)}^{2}+\lambda \mathrm{pen}\left(b\right),$$

where λ>0 is the regularisation parameter that controls the penalty strength and $\mathrm{pen}\left(b\right)$ is the penalty. For example, in the lasso method, $\mathrm{pen}\left(b\right)=||b|{|}_{1}=\overset{p}{\underset{j=1}{\sum }}\left|b_{j}\right|$, we discuss other choices below. In the case of a binary trait, the logistic regression model is usually used in which the posterior probability is modelled as:

$$P(y_{i}=1|x_{i})=\frac{\exp\left(\beta_{0}+x_{i}^{T}\beta \right)}{1+\exp\left(\beta_{0}+x_{i}^{T}\beta \right)}$$

and parameters are estimated using the penalised maximum likelihood method:

$${\hat{\beta }}_{0},\hat{\beta }=\arg\underset{b_{0}\in R,b\in R^{p}}{\max}\overset{n}{\underset{i=1}{\sum }}[y_{i}\log\left(\sigma \left(b_{0}+x_{i}^{T}b\right)\right)+\left(1-y_{i}\right)\log\left(1-\sigma \left(b_{0}+x_{i}^{T}b\right)\right)]+\lambda \mathrm{pen}\left(b\right),$$

where $\sigma \left(s\right)=\exp\left(s\right)/\left(1+\exp\left(s\right)\right)$ is the sigmoid logistic function. The multinomial logistic regression (MLR) extends the logistic model when the number of categories of the dependent variable K>2. This is the most commonly used model, as usually the considered trait has multiple categories (eye colour, skin colour, hair type, etc.). The posterior probability for the k-th category is:

$$P(y_{i}=k|x_{i})=\frac{\exp\left(\beta_{0k}+x_{i}^{T}\beta_{k}\right)}{1+\sum_{k=1}^{K-1}\exp\left(\beta_{0k}+x_{i}^{T}\beta_{k}\right)},$$

for k=1,…,K−1, where $\beta_{k}$ is a coefficients vector corresponding to the k-th category and $P\left(y_{i}=K|x_{i}\right)=1-\overset{K-1}{\underset{k=1}{\sum }}P(y_{i}=k|x_{i})$. In this model, we have K×p parameters, which are estimated using the penalised maximum likelihood method. The interaction terms $x_{i,j}\times x_{i,k}$ can be included in the above models, at the cost of a significant increase in the number of parameters. In addition to linear models, additive models are an important class of models in which, instead of the linear combination $\beta_{0}+x_{i}^{T}\beta_{}$, the combination of M non-linear base functions $\beta_{0}+\overset{M}{\underset{m=1}{\sum }}\beta_{m}h_{m}\left(x_{i}\right)$ is used. In this group, the notable approach is the MARS method (multivariate adaptive regression splines; see Section 9 in [134]) in which the functions $h_{m}$ are constructed as products of so-called hinge functions in a forward stage-wise manner. Importantly, the functions $h_{m}$ in MARS can capture non-linear dependencies as well as interactions between variables. Note that the considered model is linear in predictors $h_{m}\left(x_{i}\right)$ and is an important example of the transformation of predictors method.

Regarding regularisation in the above models, the lasso penalty $\mathrm{pen}\left(b\right)=|\left|b\right|{|}_{1}$ is the most popular choice, which was successfully used in appearance trait prediction, e.g., in prediction of human height [120] or eye colour [137]. The lasso method selects features with non-zero estimated coefficients, and the number selected depends on parameter λ>0. A small value of λ will result in a larger number of features included in the model, whereas for a larger λ, we obtain a more parsimonious model. The optimal value of λ is chosen using cross-validation or by minimising the prediction error with a validation set. An alternative to the lasso is ridge penalty $\mathrm{pen}\left(b\right)=|\left|b\right|{|}_{2}$ which, instead of performing feature selection, only shrinks the estimated parameters towards zero. The ridge penalty facilitates a reduction in the variance of the estimators, especially when the variables are highly correlated, and thus may yield an even higher accuracy for the prediction than the lasso method.

Although the lasso method has many excellent properties and high predictive power, in recent years, several modifications have been proposed in statistical and machine learning literature. For example, it has been noticed that the lasso method produces biased estimators for truly significant variables with large coefficients, and this bias does not necessarily disappear for a large sample size. To overcome this drawback, non-convex penalties, such as SCAD (smoothly clipped absolute deviation) [138] or MCP (minimax concave penalty) [139] have been proposed and effective algorithms for solving the related optimisation problems have been developed [140]. Another important line of research is focused on controlling the false discovery rate (FDR) (the expected fraction of non-significant variables that are selected for the model) instead of the much stronger control of probability that at least one non-significant variable is selected (familywise error rate). Unfortunately, the standard lasso does not control the FDR, which means that, among the selected variables, we can expect a significant portion of spurious variables. The problem is exacerbated by the fact that there is no known way of testing the significance of a specific feature based on its estimated lasso coefficient that would allow the application of one of multiple testing approaches, such as the Benjamini-Hochberg procedure [141], to control the FDR.

A notable alternative approach is the knockoff filter method [142]. It can be seen as a refinement of randomisation methods [143,144] that, by permuting the values of a studied predictor (which renders the resulting artificial predictor non-significant), creates a benchmark situation in which its usefulness can be checked. The basic idea in [142] is to construct extra variables called ’knockoff’ variables, which are noisy copies of original ones but which have a certain similar correlation structure, as they allow for FDR control when standard variable selection methods (such as lasso) are applied. Namely, the lasso method is run using both the original variables and knockoff variables (thus there are 2×p variables in total). The original variable is deemed useful when its pertaining estimated coefficient is significantly larger than that of the corresponding knockoff.

The nonconvex penalties, as well as the randomisation methods, seem to be worthwhile alternatives to the lasso method for predicting human traits. The above methods are implemented, e.g., in R software, see packages glmnet (lasso and ridge), ncvreg (MCP, SCAD), knockoff (knockoff filter), and earth (MARS method).

### 4.3. Complex Black-Box Models

The black-box model is a class of predictive models that are able to recover complex dependencies between explanatory variables and the dependent variable, including interaction terms, and which can potentially achieve higher accuracy then linear models. The main limitations are the high computational complexity, the difficulty in interpreting the model, and the necessity of parameter tuning. In this group, ensemble methods and neural networks play the leading role. The former are usually based on decision trees [145] and overcome two limitations of single trees: their instability and tendency to overfitting. The simplest approach is bagging (bootstrap aggregating) [146] in which each tree in the ensemble is trained using a bootstrap sample, i.e., a sample drawn with replacements from the original training data. In order to classify a new instance, each decision tree provides the classification for the input data. The majority vote classification is then chosen as the final prediction. In the case of regression, the predictions from individual tress are averaged. Another important class of models are random subspace methods (RSM), in which each base classifier is learnt using the randomly selected subset of variables [147,148]. One of the most successful and commonly used methods is random forest (RF), which can be seen as a combination of bagging and RSM. The RF uses a modified tree learning algorithm that selects, at each candidate split in the learning process, a random subset of the features of size m, where m is a hyper-parameter. Making m smaller helps to avoid the danger of overfitting. Nowadays, the most powerful class of ensemble methods are gradient boosting (GB) algorithms (Section 10 in [134]). In GB, the subsequent models $F_{1}\left(x\right),\ldots ,F_{M}\left(x\right)$ are learned sequentially, and the last model $F_{M}\left(x\right)$ serves as a final model. The main advantage of GB algorithms is that they are able to optimise different loss functions, depending on the considered task. The classifier in step m+1 (usually a decision tree) is learnt using current training data, in which the residuals from the previous model are treated as the current target variable (where the squared loss is considered, and the residuals are $y_{i}-F_{m}\left(x_{i}\right)$). The residuals are related to the so-called functional gradient of the loss function and, therefore, GB methods can be seen as gradient descent algorithms, which take steps in the direction of the steepest descent and converge to the minimum of the loss function. The common property of all boosting algorithms is that the current model zooms in on samples where its predecessor failed. Usually, some regularisation techniques are used in boosting algorithms to prevent overfitting. There are many versions of gradient boosting algorithms, among which XGB (extreme gradient boosting) is considered to be one of the most powerful variants [149]. The ensemble methods are controlled by different parameters, whose optimal choice may significantly improve the performance: the number of trees, the size of the random subspace (in RF and RSM), as well as the regularisation and pruning parameters.

The ensemble methods described above (RF and XGB) are often used to assess the importance of the features. The simplest approach is based on a permutation scheme and is very similar to the randomisation feature selection described above. The first method (called mean decrease accuracy) involves fitting two ensemble models (e.g., RF or XGB): the first is based on the original training data and the second is based on training data in which the values of the j-th variable are randomly permuted. The variable importance measure for the j-th variable is defined as the difference in accuracies corresponding to these two models. A large value of the difference indicates the significance of the variable. The second measure (called mean decrease impurity) is based on observing how well the given variable separates the classes. The Boruta algorithm [150], based on the above two measures, contains a testing procedure that allows the rejecting of the noisy variables. Other more sophisticated variable importance measures are also advocated for, e.g., the MCFS method [151], in which one of its major advantages is that the predictive power of each tree in the ensemble is taken into account in the measure definition.

The second important group of black-box models is artificial neural networks (ANN) [152]. The latest advances in computational and optimisation methods have made it possible to train networks with very complex architectures corresponding to large families of functions, such as convolution networks (in image recognition) and recurrent networks (in text analysis). The deep networks used today may consist of hundreds of hidden layers and can model very complex dependencies [153]. In appearance trait prediction, the feed-forward neural network is usually used. In such networks, the input signal (the vector of features for the i-th observation) is transmitted from the input layer to the output layer, which yields the prediction of the response. The hidden layers consist of artificial neurons in which the linear combination of the signals from the previous layers is computed and the signal is passed through the activation function as the input for the following layers. The models are trained using gradient algorithms (the ADAM algorithm [154] is now the state-of-the-art method) and the back-propagation algorithm is used to effectively compute the gradient of the considered risk function [153]. A number of parameters need to be tuned in ANN, such as the number of layers, the number of neurons in each layer, and the value of the learning rate. Other spectacular advances with ANN, such as variational autoencoders (VAE), which enable latent feature analysis (see [155]), are of potential interest in appearance trait prediction. For the methods described here, see R packages randomForest, xgboost, rmcfs, Boruta, and tensorflow.

### 4.4. Feature Selection

Feature selection is an essential element when building predictive models, as it prevents overfitting and allows discovering the dependency structure between variables and, in particular, recovering the features that affect the target variable. In the models described above, feature selection is usually embedded in learning algorithms. For example, in linear models as well as neural networks, selection is performed via regularisation, whereas in tree-based methods, the relevant features are selected when building the tree. However, including too many potential features may significantly increase the computational cost of fitting the model. Thus, very often in practice, there is a need to apply some fast preliminary filtering method. In the machine learning community, methods based on information theory have gained the most popularity in recent years [156]. They are fast, model free, and are able to detect non-linear dependencies and interactions between variables, as well as take into account redundancies. The basic quantity used in such methods is mutual information (MI):

$$I\left(Y,X_{k}\right)=\underset{x,y}{\sum }P\left(X_{k}=x,Y=y\right)\log\frac{P\left(X_{k}=x,Y=y\right)}{P\left(X_{k}=x\right)P\left(Y=y\right)}$$

which is a non-parametric measure of dependence between some feature $X_{k}$ and target variable Y. Moreover, analogously defined, the conditional mutual information $I(Y,X_{k}|Z)$ quantifies the dependence strength between $X_{k}$ and Y given the possibly multivariate variable Z. It is commonly used in feature selection of a new predictor $X_{k}$ when Z consists of predictors already chosen. Another important quantity used in genome-wise interaction studies (GWIS) is interaction information (*II*):

$$II\left(Y,X_{j},X_{k}\right)=I\left(\left(X_{k},X_{j}\right),Y\right)-I\left(Y,X_{k}\right)-I\left(Y,X_{j}\right)$$

which measures the interaction strength between $X_{k}$ and $X_{j}$ for the prediction of Y. The positive value of II indicates a synergistic interaction, whereas a negative value indicates redundancy. II has been successfully used in many genetic studies to detect epistasis [157,158], and also in the context of appearance trait prediction, such as human pigmentation [159]. It has been shown that the methods based on II are able to detect interactions that remain undetected by the logistic regression model [160].

The existing filters based on MI are forward sequential procedures that, in each step, add a candidate feature $X_{k}$ to the set of already selected features S. The quality of a candidate feature can be assessed using various criteria, and the representative one is CIFE (conditional infomax feature extraction) [156,161]. It adds candidate $X_{k}$, being the maximizer of $I\left(Y,X_{k}\right)+\underset{j\in S}{\sum }II\left(Y,X_{k},X_{j}\right)$. The CIFE takes into account the marginal dependence between a candidate feature and the target variable, as well as interactions between the candidate feature and the previously selected features. Methods taking into account higher order interactions are also considered [162]. In practice, it is important to decide at which step to stop the procedure of adding new candidate variables, with the possible solution based on the approximate distribution of the criterion function when a candidate feature is not significant, as proposed in [163].

## 5. The Need for a High-Throughput, Low-Input DNA Sequencing Method in Forensic Science

The limitations of DNA sequencing technologies used in the forensic field are increasingly problematic because they are hindering the implementation of new methods that can improve law enforcement and justice, and which are therefore important for the safety of society. The lack of a suitable method for generating large amounts of SNP data from degraded DNA, validated for use in forensics, was considered to be a barrier to the forensic implementation of investigative genetic genealogy, an approach that was very successful at solving a number of criminal cases [1,164]. Such a method also seems to be essential for developing next-generation tools for the DNA-based prediction of appearance traits, which requires information derived from hundreds or even thousands of SNPs. It may be argued that the optimal method for all the applications developed for forensic DNA intelligence would be whole-genome sequencing (WGS). Notably, WGS that uses high-throughput methods (massively parallel sequencing) has revolutionised the studies of ancient DNA and enabled a better understanding of human evolutionary history. Similarities between forensic genetics and palaeogenetics, especially in terms of the specificity of research material with a high content of inhibitors and small amounts of highly fragmented DNA, and the enormous success of palaeogenetics in the analysis of such samples, prompts a closer look at the methods developed in this field. Several technological advancements were crucial for the effective analysis of ancient DNA, including the very efficient extraction of short ancient DNA fragments, the implementation of the uracil-DNA glycosylase (UDG) protocol for the selective removal of damaged sections of ancient DNA, improved protocols for library preparation, and, finally, progress has also been made in high-throughput DNA sequencing [165,166]. The major advantage for ancient DNA research brought about by high-throughput sequencing technology is the ability to sequence very short DNA fragments. Research material analysed in forensic DNA laboratories is not as degraded as ancient samples, and current DNA extraction methods are efficient and effective at removing inhibitors. Therefore, the transfer of DNA analysis protocols from palaeogenomics to forensic genomics should perhaps primarily focus on library preparation methods that work well with low-input DNA. Standard library preparation protocols are optimised for large amounts of DNA and perform poorly in the case of samples containing degraded DNA. However, a number of modified protocols have been proposed to reduce the requirement for DNA inputs to be at subnanogram quantities.

One category of protocols involves library construction based on double-stranded DNA. A first protocol was described by Meyer and Kircher in 2010, and this was laborious and had limitations that resulted in the losses of ancient DNA sequences due to incompatible adapter combinations and three purification steps prior to amplification [167]. Double-stranded library preparation protocols involve the blunt-end repair of the degraded DNA fragments, the non-directional blunt-end ligation of two adapters and the fill-in of the nicks formatted between adapters and the DNA fragment [168]. A more advanced alternative of double stranded library preparation method is the protocol proposed by Carøe et al., named blunt-end-single-tube. As the name suggests, the protocol is carried out in a single tube and relies on heat denaturation instead of purification between the subsequent steps of end-repair, the ligation of double-stranded adapters to the 5′ ends, and adapter fill-in [169].

The second approach for library preparation from samples containing low amounts of degraded DNA is particularly interesting, as it implies a process of library construction based on single-stranded DNA, allowing the use of DNA that was preserved in a single-stranded state and which is considered to be more efficient compared to double-stranded approaches. The original protocol for single-stranded library preparation, although it recovered more endogenous DNA, was very expensive and laborious [170]. However, the protocol evolved to a simplified version proposed in Gansauge et al., 2017 [171]. This is a method that involves the dephosphorylation of the template DNA, the splinted ligation of a biotinylated adapter to the 3′ end, bonding to streptavidin beads, annealing an extension primer to allow the synthesis of a second strand, and the ligation of the 5′ end of a double-stranded adapter to the 3′ end of the newly synthesized strand. The authors also proposed an automated version of this protocol [172]. An interesting modification of the single-strand library preparation method was recently proposed by Kapp et al. (2021). The advantage of the method, named the Santa Cruz Reaction, relies on simplicity and cost effectiveness. The method converts single-stranded DNA into sequencing libraries using a single enzymatic reaction, enabling the simultaneous directional splinted ligation of Illumina’s P5 and P7 adapters [173]. Technological improvements in ancient DNA analysis have resulted in significant progress in sequencing efficiency. Whole-genome data from ancient hominin material were generated with an average sequence coverage of only 1.3-fold in 2010 [174] and 30-fold in 2012 [175]. The usefulness of these protocols was also confirmed in clinical research of problematic biological material, including formalin-fixed paraffin embedded tissues [176]. The future will show whether the protocols developed in palaeogenomics can be easily transferred to forensic genomics. This would undoubtedly be extremely helpful for the further development of forensic DNA intelligence methods.

## 6. Concluding Remarks

Research on the genetic architecture of natural variation in the human physical phenotype is growing in scale and involves different human populations. The genetic prediction of physical appearance traits occupies an important place in forensic research, although the available tools are limited to the least complex traits, mainly pigmentation. Notably, there are examples of using predictive methods that have been developed by the forensic community in ancient DNA research, and which have been carried out in the field of molecular anthropology [177,178,179] and in the identification of historical figures [180,181,182], which is further evidence that molecular anthropology and forensic genetics have a lot in common. Some DNA-based predictive tools developed by the forensic community have been implemented in commercial kits. The most famous ForenSeq kit allows the analysis of HIrisPlex SNPs and therefore the prediction of eye and hair colour [183]. The HIrisPlex-S variants are also available in the Ion AmpliSeq™ PhenoTrivium Panel [184]. Predicting reliable sketches in forensic science is highly desirable at the investigation stage. For this reason, there are reports of police using private companies offering services in this regard, particularly for facial appearance prediction. For example, the Snapshot Forensic DNA Phenotyping System offered by Parabon NanoLabs claims to facilitate the accurate prediction of genetic ancestry, eye colour, hair colour, skin colour, freckling, and face shape [185]. Further research offers the opportunity to better understand the evolutionary and genetic basis of human appearance traits. The prospect of future studies on the heritability of complex traits and the exploration of the importance of rare DNA variants, as well as epistatic interactions of the second and higher orders, seems interesting. The explanation of heritability will consequently enable a more reliable prediction of physical phenotype. Undoubtedly, however, the application of next-generation predictive methods, which must rely on much larger sets of predictors and more sophisticated statistical and machine learning algorithms, will require improvements in the technology of DNA polymorphism analysis used in the forensic field. Proper interpretation of the data requires knowledge of age, which is best determined via DNA methylation analysis. However, DNA methylation analysis requires the largest amounts of DNA, so in studying biological traces for intelligence purposes, it would be beneficial to develop more sensitive age prediction methods. The application of novel predictive approaches will also require answers to important ethical questions arising from the use of high-throughput DNA analysis methods.

## Figures and Tables

**Table 1:** Examples of various approaches proposed for genetic prediction of physical traits.

Physical Trait	Statistical Model	Number of Predictors in the Model	Prediction Accuracy Parameters	Ref.
Eye colour	Multinomial logistic regression (IrisPlex) ^1^	6 SNPs	AUC_brown_ = 0.93 ^2^ AUC_intermediate_ = 0.72 AUC_blue_ = 0.91	[98]
	Likelihood ratio	4 SNPs	LR_light-dark_ depends on genotypes	[99]
	Multiple linear regression	3 SNPs	R^2^ = 0.764	[100]
	No statistical model, classification based on genotypes	6 SNPs	Overall classification success rate (blue–green–brown): 98.94%	[101]
	Likelihood ratio	6 SNPs	LR_light-dark_ depends on genotypes AUC_light-dark_ = 0.925	[102]
	Bayesian naïve classifier (Snipper)	23 SNPs	Classification success rate: blue = 98.27%, green-hazel = 97.81% brown = 96.67%.	[103]
	Multiple response classification tree	4 SNPs	Classification success rate: blue = 89% intermediate = 46% brown = 94%	[104]
	No statistical model, prediction based on genotypes	5 SNPs	Overall classification success rate (blue–green–brown): 97.64%	[105]
Hair colour	Multinomial logistic regression + prediction guide (HIrisPlex) ^1^	22 SNPs	Classification success rate: AUC_blond_ = 0.81 AUC_brown_ = 0.82 AUC_black_ = 0.87 AUC_red_ = 0.93	[106]
	Bayesian naïve classifier (Snipper)	12 SNPs	Classification success rate: blond = 92.3% brown = 76.7% black = 74.6% red = 85% Sex-related prediction accuracy differences noted	[107]
	Multinomial logistic regression	270 SNPs	AUC_blond_ = 0.74 AUC_brown_ = 0.68 AUC_black_ = 0.86 AUC_red_ = 0.86	[37]
Skin colour	Multiple linear regression, including interaction	3 SNPs	R^2^ = 0.496	[100]
	No statistical model, classification based on genotypes	5 SNPs	Overall classification success rate (dark–medium–light): 62% 38% of results inconclusive	[105]
	Bayesian naïve classifier (Snipper)	10 SNPs	AUC_white_ = 0.999AUC_intermediate_ = 0.803 AUC_black_ = 0.966	[108]
	Multinomial logistic regression (HIrisPlex-S) ^1^	36 SNPs	AUC_light_ = 0.97 AUC_dark_ = 0.83 AUC_dark-black_ = 0.96 or AUC_very-pale_ = 0.74 AUC_pale_ = 0.72 AUC_intermediate_ = 0.73 AUC_dark_ = 0.87 AUC_dark-black_ = 0.97	[109]
	Multiple linear regression	9 SNPs	R^2^ = 0.65	[110]
Freckles	Binomial logistic regression	34 SNPs + sex	AUC_freckled_ = 0.809	[111]
	Multinomial logistic regression	20 SNPs + sex	AUC_non-freckled_ = 0.754 AUC_freckled_ = 0.657 AUC_heavily-freckled_ = 0.792	[112]
Hair loss	Binomial logistic regression	20 SNPs	AUC_bald_ = 0.66 AUC_bald_ = 0.76 in men ≥ 50 years old	[113]
	Binomial logistic regression	14 SNPs	AUC_early-onset baldness_ = 0.74	[114]
	Polygenic scores (weighted allele sums)	261 autosomal SNPs; 70 X chromosomal SNPs	AUC_severe baldness_ = 0.748 (autosomal SNPs)AUC_severe baldness_ = 0.621 (X chromosome SNPs) With autosomal and X SNPs + age included in the model: AUC_severe baldness_ = 0.79; AUC_moderate baldness_ = 0.70; AUC_slight baldness_ = 0.61	[52]
Hair shape	Binomial logistic regression	3 SNPs	AUC_straight_ = 0.62	[115]
	Binomial and multinomial logistic regression	32 SNPs in binomial modelor33 SNPs in multinomial model	AUC_straight_ = 0.66 in Europeans AUC_straight_ = 0.79 in non-Europeans or AUC_straight_ = 0.67 in Europeans AUC_wavy_ = 0.60 in Europeans AUC_curly_ = 0.60 in Europeans AUC_straight_ = 0.80 in non-Europeans AUC_wavy_ = 0.61 in non-Europeans AUC_curly_ = 0.74 in non-Europeans	[116]
Hair greying	Binary and multi-class neural network	10 SNPs + age and sex in binary model or 12 SNPs + age and sex in multi-class model	AUC_greying_ = 0.87 (mostly based on age)or AUC_no greying_ = 0.86 AUC_mild greying_ = 0.79 AUC_severe greying_ = 0.86	[66]
Height	Polygenic scores (weighted allele sums)	54 SNPs	AUC_tall stature_ = 0.65	[117]
	Polygenic scores (weighted allele sums)	180 SNPs	AUC_tall stature_ = 0.75	[118]
	Polygenic scores (weighted allele sums)	689 SNPs	AUC_tall stature_ = 0.79	[119]
	L_1_-penalized regression (LASSO)	>20,000 SNPs	r = 0.64	[120]
Face	Partial least squares regression	Genomic ancestry (68 DNA variants) + sex + 24 SNPs	Genomic ancestry explains 9.6% of the total facial variation; sex independently from ancestry explains 12.9%; SNPs make a small contribution to improving facial distinctiveness	[82]
	Ridge regression	Genomic ancestry (1000 genomic Principal Components) + sex, BMI and age	Genomic ancestry and sex explain large proportion of the predictive accuracy of the model; age and BMI improve the accuracy of the model	[121]
	Simple quantitative method (principal component analysis and partial least square analysis used to extract new face traits)	277 SNPs	SSA statistic ^3^: no difference between SNP-based prediction and random predictions in females; SNP-based predictions significantly better than random predictions in males	[93]

^1^ SNaPshot and MPS forensically validated genetic tests for data collection available; ^2^ AUC—area under the ROC (receiver operating characteristic) curve, describes the general performance of the model, 1 means perfect prediction and 0.5 means random assignment; ^3^ SSA—a shape similarity statistic (shape space angle) developed to measure the angle between two shapes in the 3D face modelling data space.
